# Selective uterine artery embolization is a valid adjuvant treatment of choriocarcinoma: a case report and literature review

**DOI:** 10.3389/fonc.2024.1479603

**Published:** 2024-11-12

**Authors:** Yuying Chen, Tingting Sun, Linjing Yuan, Yucong Huang, Aligu Yusufu, Yang Zhang, Xiaoyan Zhang, Shasha He, Yufeng Ren, Shuzhong Yao, Guofen Yang

**Affiliations:** ^1^ Department of Gynecology, Sun Yat-sen University First Affiliated Hospital, Guangzhou, China; ^2^ Department of Radiation Oncology, Sun Yat-sen University First Affiliated Hospital, Guangzho, China

**Keywords:** choriocarcinoma, primary cervical choriocarcinoma, selective uterine artery embolization, fertility retention, misdiagnosis

## Abstract

**Background:**

Cervical choriocarcinoma is an extremely rare malignancy that is often misdiagnosed due to its nonspecific symptoms, such as vaginal bleeding.

**Case report:**

A 39-year-old female presented to the emergency department of the First Affiliated Hospital of Sun Yat-sen University with vaginal bleeding and a serum β-human chorionic gonadotropin (β-HCG) level of 229,386 mIU/mL. Initially, she was misdiagnosed with cervical pregnancy and subsequently underwent selective uterine artery embolization and cervical mass excision. However, pathological examination revealed the diagnosis of cervical choriocarcinoma.

**Conclusion:**

This case highlights the propensity for misdiagnosis of cervical choriocarcinoma. Selective uterine artery embolization proves to be an efficient measure to manage hemorrhage and potentially avoid unnecessary hysterectomy.

## 1 Introduction

Choriocarcinoma is a rare malignant trophoblastic tumor that happens in one in 40,000 pregnancies in Europe and North America, while 9.2 and 3.3 per 40,000 pregnancies in Southeast Asia and Japan (1). Choriocarcinoma usually develops in the uterine cavity and is associated with a concurrent or previous pregnancy (2). Extrauterine choriocarcinomas are rare, with the uterine cervix being the most common site of occurrence (3). Prompt and accurate diagnosis of cervical choriocarcinoma (CC) is crucial for effective early treatment. However, its clinical manifestations, such as irregular vaginal bleeding, and examination findings, including elevated β-human chorionic gonadotropin (β-HCG) level and ultrasound indications of increased blood flow to the uterus or adrenal area, are often nonspecific. Consequently, CC is frequently misdiagnosed as cervical ectopic pregnancy, cervical fibroids, cervical polyps, and so forth.

Choriocarcinoma is a highly chemo-sensitive malignancy. Therefore, for the treatment of patients with uterine choriocarcinoma, single-agent chemotherapy is effective for low-risk patients, while multi-agent chemotherapy is recommended for high-risk patients. Both approaches can achieve a satisfactory remission rate and overall survival, with the potential for fertility preservation (4–6). However, in cases of CC, uncontrollable vaginal bleeding can necessitate an emergent hysterectomy, which compromises the patient’s fertility (7, 8). As most of the CC patients were women in their childbearing age, effective reproductively protective treatment has become an urgent clinical need.

Due to the rarity of CC, only a limited number of cases have been reported to date. In this article, we present a case of CC in which reproductive function was successfully preserved through selective uterine artery embolization. Additionally, we reviewed the existing literature to summarize the clinical characteristics and effective treatments for CC, aiming to enhance understanding of the disease among patients and gynecologists, thereby facilitating informed treatment decisions.

## 2 Case presentation

A 39-year-old Chinese female, gravida 2, para 2, presented to the emergency department of the First Affiliated Hospital of Sun Yat-sen University for vaginal bleeding on 7 March 2021. Her obstetric history includes a cesarean section in 2013 for her first pregnancy and a spontaneous full-term vaginal delivery on 17 February 2020 for her second pregnancy. After her most recent delivery, she experienced one normal menstrual cycle in July 2020 but had no regular menstruation thereafter. The vaginal bleeding began on 22 January 2021 and became severe on 7 March 2021. Her blood test revealed a β-HCG level of 229,386 mIU/mL and a hemoglobin level of 110g/L when admitted. Pelvic examination identified multiple blood clots in the vagina, and a barrel-shaped cervix, with a mass of approximately 6 cm × 5 cm protruding from inside of the cervix. Cervical pregnancy was initially suspected.

**Figure 1:**
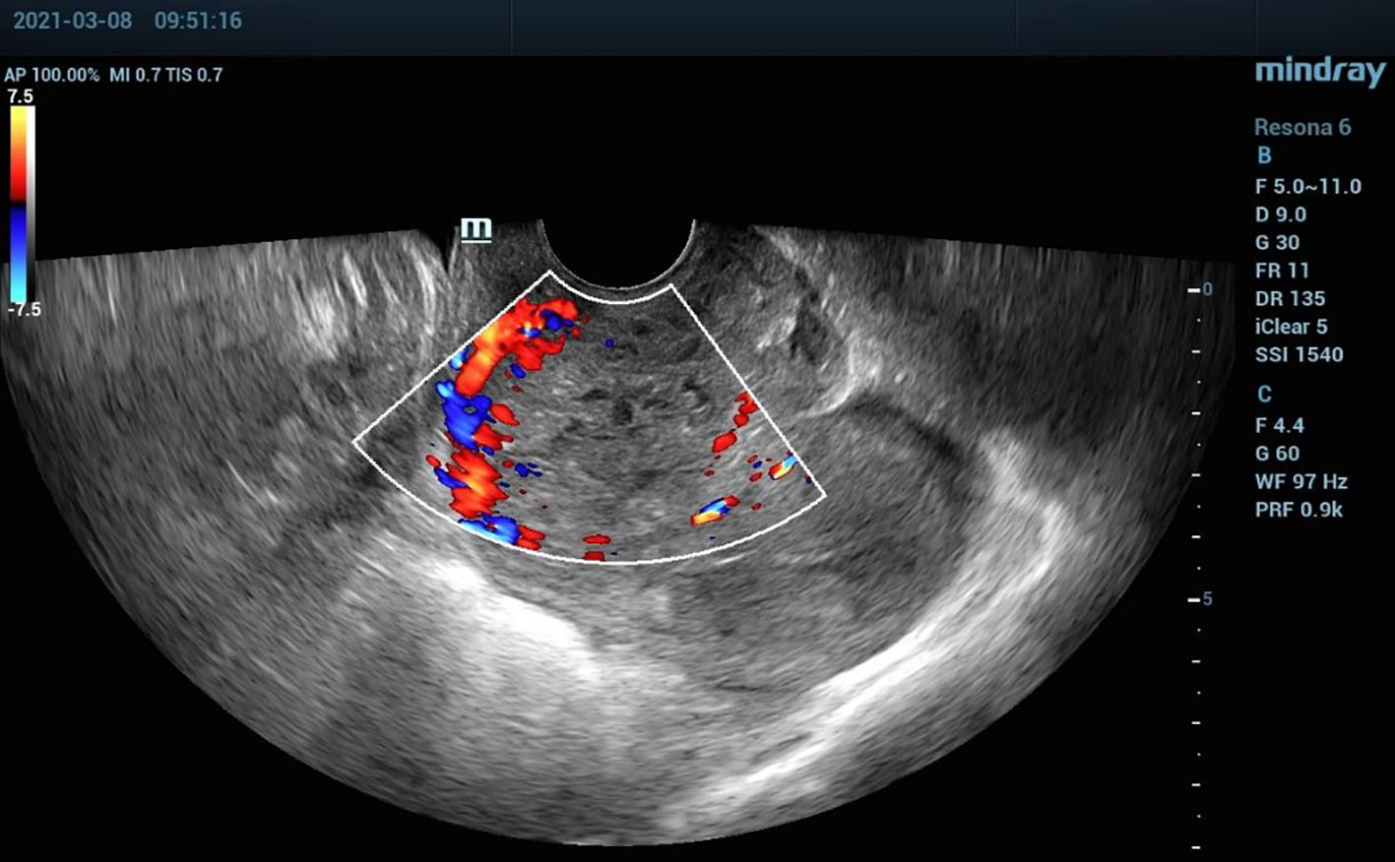
Ultrasound color Doppler image of the cervix, showing a highly vascularized zone.

A transvaginal ultrasound scan demonstrated a mass of approximately 54 mm × 42 mm within the cervical canal (**Figure 1**), which was considered to be cervical pregnancy with a high possibility of implantation. The chest computed tomography (CT) result was negative. To mitigate the risk of uncontrolled bleeding during the removal of the pregnancy tissue, selective uterine artery embolization was performed primarily and 40 mg methotrexate was injected into the uterine arteries simultaneously. An additional 30 mg methotrexate was given via intramuscular injection over the next 2 days. On the second day after the uterine artery embolization, the cervical mass was removed transvaginally, with an estimated blood loss of approximately 30 mL. Postoperatively, her serum β-HCG level decreased to 19,281 mIU/mL, and no metastases were found through the positron emission tomography–CT (PET-CT) scan or the pelvic magnetic resonance imaging scan.

**Figure 2:**
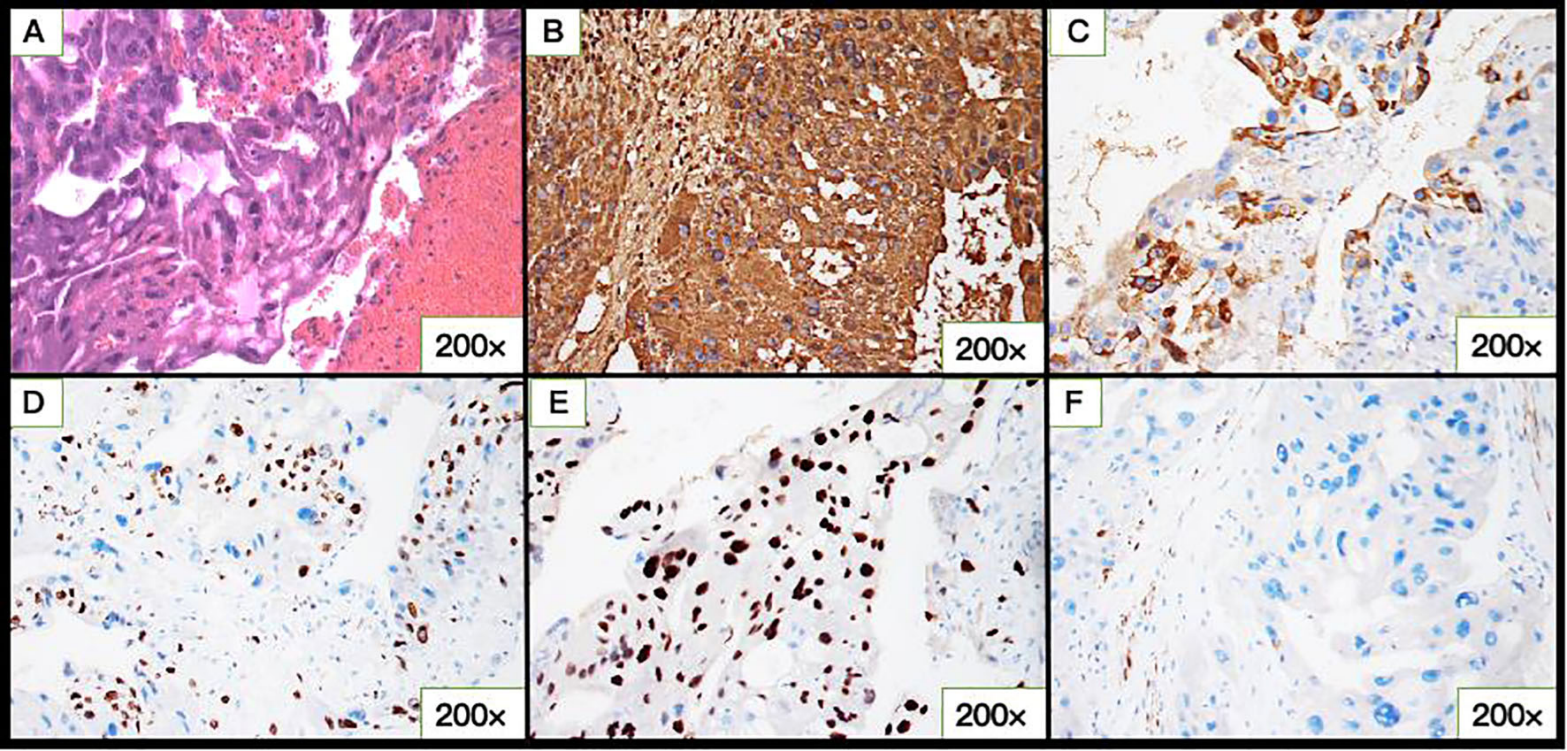
Microscopic and immunohistochemical of cervical choriocarcinoma (CC). **(A)** Hematoxylin-eosin–stained section showing atypical cytotrophoblastic cells and syncytiotrophoblastic cells with pronounced cytologic atypia and areas of hemorrhage in the tumor bulk. **(B)** hCG, **(C)** HPL, **(D)** Ki-67, **(E)** P-57, and **(F)** PLAP expression in CC.

The pathological examination revealed tissue consisting of infiltrative and destructive nest-like multinucleated heteromorphic cells, cytotrophoblastic cells, syncytiotrophoblastic cells, and massive hemorrhagic lesion with no evidence of chorionic villi (**Figure 2A**). Immunohistochemical staining of the tumor cells was positive for HCG, P57, and HPL, negative for PLAP, and the Ki-67 index was 30% positive (**Figures 2B–F**). These findings further supported the diagnosis of CC.

**Figure 3:**
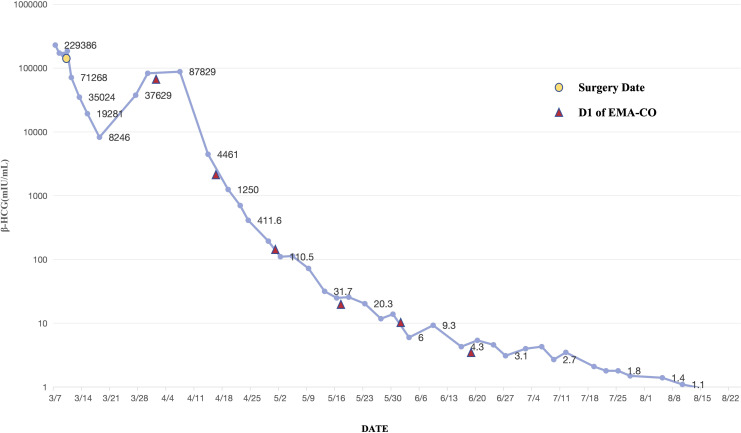
The trend of serum β-HCG level of the patient.

According to the Federation International of Gynecology and Obstetrics (FIGO) staging and classification for gestational trophoblastic neoplasia and the WHO scoring system based on prognostic factors, this patient was diagnosed with Stage I:12. Consequently, the first cycle of chemotherapy, consisting of a combination of etoposide, methotrexate, actinomycin D, cyclophosphamide, and vincristine (EMA-CO), was initiated on April 1. After five cycles of chemotherapy, her β-HCG level declined to normal range (**Figure 3**). The patient received another three cycles of chemotherapy subsequently. Her menstrual cycle resumed in September 2021. As of 3 April 2024, follow-up ultrasound examinations and β-HCG tests have revealed no abnormalities. The patient is satisfied with the treatment outcomes and is glad that the fertility has been preserved.

## 3 Discussion

Choriocarcinoma is a rare, malignant form of gestational trophoblastic tumor, which can present as either intrauterine or extrauterine disease. Extrauterine choriocarcinomas can occur in the fallopian tubes, vagina, vulva, cervix, or pelvic region and are relatively common in the cervix. We identified 100 previously reported cases of CC by reviewing studies published between 1952 and 2024 in the PubMed database (**Supplementary Table S1**). After excluding cases lacking detailed information, 24 cases were included in the final analysis (**Table 1**; **Supplementary Table S2**). In our analyzed cohort, 33.33% (8 of 24) of patients had tumors larger than 5 cm. Metastasis was observed in 37.50% (9 of 24) of the CC cases, with the lung being the most common site of metastasis, followed by the liver. The mortality rate of metastasized CC was 66.67% (6 of 9). The main treatment was combined chemotherapy, including EMA-CO, MAC (methotrexate, actinomycin D, and cyclophosphamide), and the combination of 5-Fluorouracil and actinomycin-D (preferred in China).

**Table 1:** The clinical characters of 24 cervical choriocarcinoma cases.

ID	Age	FIGO	Preceding pregnancy	Interval	HCG(mIU/mL)	Tumor (cm)	Metastasis	Chemotherapy	Prognosis	Derivation
1	36	I:12	term	>12months	>10^5^	>5	none	Carboplatin and 5-Fluorouracil. 5-Fluorouracil and actinomycin-D	survived	The Chinese-German Journal of Clinical Oncology 2009;8(6):366-8
2	46	I:11	abortion	>12months	>10^5^	<3	none	Methotrexate and actinomycin-D	survived (>2 years)	Asia-oceania j. Obstet. Gynaecol 1988;14(3):285-292
3	43	I:8	term	>12months	<10^3^	3~4	brain, lung	Methotrexate (orally)	survived (>4 years)	Asia-oceania j. Obstet. Gynaecol 1988;14(3):285-292
4	29	I	abortion	>12months	unknown	unknown	lung	None	died (<15 months)	Asia-oceania j. Obstet. Gynaecol 1988;14(3):285-292
5	29	I:8	mole	9months	>10^5^	>5	none	Actinomycin-D	survived (>20 months)	Acta obstet gynecol scand 1992;71:479-81
6	55	I:6	abortion	>12months	<10^3^	<3	brain, liver, lung	Methotrexate, actinomycin D, and cyclophosphamide	died (<2 years)	J.obstet.gynaecol.res 1996;22(5): 437-441
7	38	I:9	term	4months	>10^5^	>5	none	Methotrexate, actinomycin-D, and cyclophosphamide	survived (>2 year)	Gynecologic oncology 1997;64:274-278
8	54	I:10	term	>12months	>10^4^	3~4	none	EMA-CO	survived	Gynecologic oncology 2003;90(3):667-9
9	32	IV	no sexual relations	unknown	>10^5^	>5	liver, lung	EMA-CO.EP-EMA.5-Fluorouracil and Taxotere	died (<1 year)	Gynecol oncol 2005;98(1):146-50
10	55	III	–	unknown	>10^4^	3~4	lung	EMA-CO	died (< 3 years)	Gynecol oncol 2006;101(2):346-8
11	46	I:9	abortion	>12months	>10^4^	3~4	none	Actinomycin-D, cyclophosphamide and methotrexate	survived (>17 years)	Int j gynecol cancer 2007;17(3):715-9
12	21	I:11	mole	>12months	>10^3^	>5	none	Actinomycin-D, cyclophosphamide and methotrexate. Adriamycin and 5-Fluorouracil.	survived (>16 years)	Int j gynecol cancer 2007;17(3):715-9
13	35	III:7	abortion	>12months	>10^3^	<3	lung	Actinomycin-D, cyclophosphamide and methotrexate.5-Fluorouracil.	survived (>14 years)	Int j gynecol cancer 2007;17(3):715-9
14	30	III:7	term	12months	>10^3^	3~4	lung	Actinomycin-D, cyclophosphamide and methotrexate	survived (≈6 years)	Int j gynecol cancer 2007;17(3):715-9
15	43	I:8	term	7~12	>10^3^	>5	none	Methotrexate	survived (>1 year)	Int j gynecol cancer 2007;17(4):921-5
16	47	I	–	>12months	<10^3^	>5	none	Actinomycin-D	survived (>9 months)	Asian pac j cancer prev 2007;8:642-4
17	32	I	–	>12months	>10^4^	unknown	none	EMA-CO	survived	Eur j obstet gynecol reprod biol 2008;141(1):87-8
18	54	IV	–	unknown	>10^4^	unknown	liver, lung, lymph nodes	Cisplatin, etoposide, and bleomycin. Cisplatin, vinblastine, and ifosfamide.	died (< 1 year)	J Clin Oncol 2011;29(11):e301-2
19	30	I:8	abortion	>12months	>10^4^	3~4	none	EMA-CO	survived	J res med sci 2013;18(10):914-7
20	38	I:5	abortion	>12months	<10^3^	<3	none	none	survived (>1 year)	Arch iran med 2014;17(11):783-5
21	31	I:9	term	>12months	>10^4^	3~4	none	EMA-CO	survived	J obstet gynaecol res 2015;41(8):1291-4
22	52	I	term	>12months	unknown	<3	none	–	–	Int j gynecol pathol 2017;36(4):323-7
23	45	IV	–	unknown	>10^4^	>5	liver, bone, lung	Paclitaxel and carboplatin. EMA-CO.	died (<1 year)	J obstet gynaecol 2018;38(2):289-90
24	41	I:6	abortion	9months	>10^3^	3~4	none	Chemotherapy (no details on drugs)	survived	Chirurgia (Bucur). 2023;118(2):202-7.

*EMA-CO: Combination of etoposide, methotrexate, actinomycin D, cyclophosphamide and vincristine.

*FIGO: Federation International of Gynecology and Obstetrics.

*EP-EMA: Combination of etoposide, Cisplatin, methotrexate and actinomycin D.

Due to its atypical presentation and the lack of characteristic symptoms, CC is often misdiagnosed, leading to delays in chemotherapy, distal metastasis, and potentially ineffective treatment. In our case, the patient was initially misdiagnosed as cervical pregnancy. Among the 24 cases analyzed, 10 were misdiagnosed (**Supplementary Table S3**), 3 of 24 were ambiguously classified as “cervical tumor,” while only 4 cases received an accurate primary diagnosis. For the remaining seven cases, primary diagnosis information was unavailable (**Table 1**; **Supplementary Table S2**). Ectopic pregnancy, particularly cervical pregnancy, was the most common misdiagnosis for CC (**Supplementary Table S3**), which aligns with findings from previous studies (2).

The diagnosis of CC can be challenging (9). The diagnostic criteria of primary choriocarcinoma of the cervix include the absence of the lesions in the uterine cavity, pathologic confirmation of the disease, exclusion of a molar pregnancy, and exclusion of the coexistence of a normal intrauterine pregnancy (10). Despite meeting all these criteria, an accurate diagnosis was not initially possible in our case. Additionally, among the 24 reported cases of CC, vaginal bleeding was the most common symptom, occurring 22 of 24 cases. However, vaginal bleeding is a non-specific symptom, making the accurate diagnosis of CC difficult (9). As a result, pathological diagnosis remains the definitive criterion for confirming CC. However, atypical pathology types would limit the validity of diagnosis. In **Supplementary Tables S2**, **S3**, four atypical pathology types were identified: clear-cell adenocarcinoma with choriocarcinomatous component, non-keratinizing squamous cell carcinoma with choriocarcinoma differentiation, clear-cell carcinoma of the cervix with choriocarcinomatous differentiation, and choriocarcinomas with metaplastic transformation from adenocarcinoma. These diagnoses were primarily based on biopsy findings, which can easily lead to misdiagnosis. Thus, molecular diagnosis of choriocarcinoma should be interpreted with caution in future cases.

In this case, bilateral uterine artery embolization was performed as a precautionary measure. However, in three other published cases (11–13), uterine artery embolization was typically used as a treatment for massive hemorrhage rather than a preventive approach. In one case, uterine artery embolization was required three times to control severe vaginal bleeding (11). In our case, a small dose of methotrexate was injected into the uterine artery during embolization to inhibit and destroy some of the trophoblastic cells, which differs from the approaches used in previous cases (11–13). Specifically, one published case used different drugs during embolization (12), and no drugs were used in other two published cases (11, 13). In our case, the primary lesion was removed after the embolization, whereas in two published cases, the lesion was not excised (11, 12), and one case ultimately required a hysterectomy (13). Administrating a small dose of chemotherapy drugs during embolization, combined with timely removal of the primary lesion, effectively prevented the need for repeated artery embolization to control massive vaginal bleeding and ensured the success of subsequent treatment.

Chemotherapy is the primary treatment for choriocarcinoma, with surgery and other therapies serving as adjuncts. The treatment plan is tailored according to FIGO stage, patient age, fertility considerations, and financial circumstances. In our case, the patient was diagnosed with Stage I:12, high-risk CC, for which EMA-CO or 5-Fu-based combination chemotherapy regimen was recommended (6, 14). The patient received a total of eight cycles of EMA-CO regiment, with her serum β-hCG level returning to normal after five cycles. According to previous studies, for high-risk gestational trophoblastic disease, the complete remission rate and overall-survival rate of EMA-CO regimen were both above 90% after initial treatment (5). The most common side effect of EMA-CO was myelosuppression (15). However, with the granulocytic stimulator support and prophylactic antiemetic therapy, the dose intensity of EMA-CO regimens can be maintained (15, 16).

Although choriocarcinoma is a malignant disease, patients generally have promising progression-free survival rates (14). However, many patients underwent hysterectomy due to the massive vaginal bleeding of CC, or to prevent metastasis, resulting in the loss of fertility. 79.17% of the 24 CC patients (19 of 24) were of reproductive age (15–49 years), yet 91.67% (22 of 24) cases lost their uterus as part of their treatment (**Supplementary Table S2**). Selective uterine artery embolization is an effective treatment option that can efficiently control blood loss and make it possible to remove the lesion without the need for hysterectomy. In our case, although the patient was initially misdiagnosed with cervical pregnancy, timely uterine artery embolization allowed for the preservation of her uterus.

A potential limitation of our study is that cases reported in languages other than English may not have been included, as they were not accessible through our search. Despite this, we conducted a detailed analysis of the clinicopathological characteristics and treatments of 24 cases, which may aid in improving the diagnosis and management of CC. Additionally, we identified four unique mixed pathology types, the treatment of which warrants further investigation and discussion.

## 4 Conclusion

In conclusion, CC is a rare but highly chemo-sensitive tumor. However, due to its nonspecific symptoms, it is frequently misdiagnosed. Most of the CC patients are women of childbearing age, making fertility preservation a key concern. Selective uterine artery embolization is an effective fertility-preserving treatment that controls blood loss, allows for lesion removal, and provides time for biopsy, thereby reducing the need for unnecessary hysterectomy.

## Data Availability

The original contributions presented in the study are included in the article/**Supplementary Material**. Further inquiries can be directed to the corresponding author.
