# Supplementary material for: Selective uterine artery embolization is a valid adjuvant treatment of choriocarcinoma: a case report and literature review
Source: Front Oncol. 2024 Nov 12;14:1479603. doi: 10.3389/fonc.2024.1479603 (PMC11588632; doi:10.3389/fonc.2024.1479603)
Supplement: Supplementary file 1 [file DataSheet1.docx]

**Supplementary Table 1.** 100 reported cases of cervical choriocarcinoma.

| **Author/language** | **Case(s)** | **Derivation** |
| --- | --- | --- |
| **Rashbaum/English** | **13** | Am J Obstet Gynecol 1952;64:451–5 |
| **Saito/ Japanese** | **18** | World Obstet Gynecol 1965;17:459–84 |
| **Minegishi/ Japanese** | **6** | Sanfujinka Jissai 1965;14:763–7 |
| **Ooguchi/ Japanese** | **7** | J Jpn Obstet Gynecol Soc 1966;18:1083–92 |
| **Koga/ Japanese** | **1** | J Jpn Obstet Gynecol Soc 1966;13:245–9 |
| **Danek/ Italian** | **1** | Minerva Ginecol 1969;21:1707–11 |
| **Momose/ Japanese** | **1** | Obstet Gynecol Ther 1970;21:468–71 |
| **Pavlica/ German** | **1** | Zentralbl Gynakol 1971;93:72–4 |
| **Tsukamoto/ English** | **1** | Gynecol Oncol 1980;9:99–107 |
| **Tripathi/ English** | **1** | Br J Obstet Gynaecol 1982;89:267–9 |
| **Meriah/ French** | **1** | J Gynecol Obstet Biol Reprod (Paris) 1983;12:519–24 |
| **Martin/ English** | **1** | Am J Obstet Gynecol 1983;147:343–4 |
| **Bogdanowicz/ Polish** | **1** | Ginekol Pol 1984;55:527–30 |
| **Bhalla/ English** | **1** | Indian J Pathol Microbiol 1987;30:51–3 |
| **Tsai, Y. S./ English** | **4** | Asia Oceania J Obstet Gynaecol. 1988 Sep;14(3):285-92. |
| **Ben-Chetrit/ English** | **1** | Am J Obstet Gynecol 1990;163:1161–3 |
| **Lee, J. D./ English** | **1** | Acta Obstet Gynecol Scand. 1992 Aug;71(6):479-81. |
| **Heyn/ German** | **1** | Geburtshilfe Frauenheilkd 1993;53:498–500 |
| **Herts/ English** | **1** | J Ultrasound Med 1993;12:59–62 |
| **Abboud/ French** | **1** | J Gynecol Obstet Biol Reprod (Paris) 1994;23:149–51 |
| **al Hassani/ English** | **1** | Trop Geogr Med 1995;47:308–9 |
| **Morimura, Y./ English** | **1** | J Obstet Gynaecol Res. 1996 Oct;22(5):437-41. |
| **Yahata, T./ English** | **1** | Gynecol Oncol. 1997 Feb;64(2):274-8. |
| **Lema/English** | **1** | East Afr Med J 1997;74:600–2 |
| **Baykal, C./ English** | **1** | Gynecol Oncol. 2003 Sep;90(3):667-9. |
| **Roopnarinesingh/English** | **1** | Ir Med J 2004;97:147–8 |
| **Maesta, I. / English** | **1** | Gynecol Oncol. 2005 Jul;98(1):146-50. |
| **Peko/French** | **1** | Med Trop (Mars) 2005;65:498 |
| **Pavelka J.C. / English** | **1** | Gynecol Oncol. 2006 May;101(2):346-8. |
| **Fu, Y. / English** | **4** | Int J Gynecol Cancer. 2007 May-Jun;17(3):715-9. |
| **Kairi-Vassilatou, E. / English** | **1** | Int J Gynecol Cancer. 2007 Jul-Aug;17(4):921-5. |
| **Chumworathayi, B. / English** | **1** | Asian Pacific journal of cancer prevention : APJCP, 2007. 8(4): p. 642-644. |
| **Frati, A. / English** | **1** | Eur J Obstet Gynecol Reprod Biol. 2008 Nov;141(1):87-8. |
| ***Wang, Y./English** | **1** | The Chinese-German Journal of Clinical Oncology, 2009. 8(6): p. 366-368. |
| **Chandacham, A. / English** | **1** | Journal of the Medical Association of Thailand = Chotmaihet thangphaet, 2009. 92(1): p. 120-123. |
| **Longo, R. / English** | **1** | J Clin Oncol. 2011 Apr 10;29(11):e301-2. |
| **Sorbi, F. / English** | **1** | J Res Med Sci. 2013 Oct;18(10):914-7. |
| **Mitrovic, S. / English** | **1** | Arch Iran Med. 2014 Nov;17(11):783-5. |
| **Park, M. / English** | **1** | J Obstet Gynaecol Res. 2015 Aug;41(8):1291-4. |
| **Mukonoweshuro, P. / English** | **1** | Int J Gynecol Pathol. 2017 Jul;36(4):323-327. |
| **Hwang, D. W. / English** | **1** | J Obstet Gynaecol. 2018 Feb;38(2):289-290. |
| **Wang, X./English** | **12** | Orphanet J Rare Dis. 2021;16(1):480. |
| **Karafezieva, B. V./English** | **1** | Chirurgia (Bucur). 2023;118(2):202-7. |

**Supplementary Table 2.** Clinical details of the 24 CC cases.

| **Relevant factors** | | | **Number** | **Percentage (%)** |
| --- | --- | --- | --- | --- |
| **Pathology** | Choriocarcinoma | | 20 | 83.33 (20/24) |
|  | Others | | 4 | 16.67 (4/24) |
| **Primary diagnosis** | Misdiagnosed | | 10 | 41.67 (10/24) |
|  | Correctly diagnosed | | 4 | 16.67 (4/24) |
|  | Ambiguous diagnosed | | 3 | 12.5 (3/24) |
|  | Unknown | | 7 |  |
| **Age** | ＜40 | | 12 | 50.00 (12/24) |
|  | ≥40 | | 12 | 50.00 (12/24) |
|  | Mole | | 2 | 8.33 (2/24) |
| **Preceding pregnancy** | Abortion | | 8 | 33.33 (8/24) |
|  | Term | | 8 | 33.33 (8/24) |
|  | Unknown | | 6 |  |
| **Interval**（months） | ＜4 | | 0 | 0 |
|  | 4-6 | | 1 | 4.17 (1/24) |
|  | 7-12 | | 4 | 16.67 (4/24) |
|  | ＞12 | | 15 | 62.50 (15/24) |
|  | Unknown | | 4 |  |
| **HCG**（mIU/mL） | ≤10^3^ | | 4 | 16.67 (4/24) |
|  | ＞10^3^-10^4^ | | 5 | 20.83 (5/24) |
|  | ＞10^4^-10^5^ | | 8 | 33.33 (8/24) |
|  | ＞10^5^ | | 5 | 20.83 (5/24) |
|  | Unknown | | 2 |  |
| **Tumor size**（cm） | ＜3 | | 5 | 20.83 (5/24) |
|  | 3-4 | | 8 | 33.33 (8/24) |
|  | ≥5 | | 8 | 33.33 (8/24) |
|  | Unknown | | 3 |  |
| **Metastases** | Lung | | 4 | 16.67 (4/24) |
|  | Spleen，kidney | | 0 | 0 |
|  | Gastrointestinal | | 0 | 0 |
|  | Brain，liver | | 5 | 20.83 (5/24) |
|  | None | | 15 | 62.50 (15/24) |
| **FIGO** |  | Score |  |  |
|  | I | 0-6 | 3 | 12.50 (3/24) |
|  |  | 7-12 | 10 | 41.67 (10/24) |
|  |  | ≥13 | 0 | 0 |
|  |  | Unknown | 5 |  |
|  | II |  | 0 | 0 |
|  | III | 0-6 | 0 | 0 |
|  |  | 7-12 | 2 | 8.33 (2/24) |
|  |  | ≥13 | 0 | 0 |
|  |  | Unknown | 1 |  |
|  | IV | Unknown | 3 |  |
| **Chemotherapy** | Single drug | | 4 | 16.67 (4/24) |
|  | Two or more drugs | | 16 | 66.67 (16/24) |
|  | None | | 2 | 8.33 (2/24) |
|  | Unknown | | 2 |  |
| **Reproductive function** | Lost | | 22 | 91.67 (22/24) |
|  | Preserved | | 2 | 8.33 (2/24) |

**Supplementary Table 3.** The misdiagnosis of the 24 CC cases.

| **Misdiagnosis** | | **Number of cases** |
| --- | --- | --- |
| **ectopic pregnancy** | cervical pregnancy | 2 |
|  | cesarean section scar pregnancy | 1 |
|  | location unknown | 1 |
| **squamous cell carcinoma** | | 2 |
| **cervical polyp** | | 1 |
| **adenocarcinoma** | | 1 |
| **clear cell carcinoma** | | 1 |
| **dysfunctional uterine hemorrhaging** | | 1 |
